# High temporal resolution records of outdoor and indoor airborne microplastics

**DOI:** 10.1007/s11356-022-24935-0

**Published:** 2023-01-04

**Authors:** Lucy C. Boakes, Ian R. Patmore, Chiara E. P. Bancone, Neil L. Rose

**Affiliations:** Environmental Change Research Centre, Department of Geography, University College London, Gower Street, London, WC1E 6BT UK

**Keywords:** Airborne microplastics, Atmospheric pollution, Burkard trap, Hourly resolution, Urban environments

## Abstract

There is increasing concern regarding airborne microplastics, but to date, studies have typically used coarse interval sampling (a day or longer) to generate deposition and concentration estimates. In this proof-of-concept study, we used a Burkard volumetric spore trap (intake 10 L min^−1^; recording airborne particulates onto an adhesive-coated tape moving at 2 mm hr^−1^) to assess whether this approach has potential to record airborne microplastics at an hourly resolution, thereby providing detailed diurnal patterns. Simultaneous sampling at outdoor and indoor locations at rural and urban sites showed clear daily and weekly patterns in microplastic concentrations which may be related to people and vehicle movement. Indoor residential concentrations of suspected microplastics were the highest (reaching hourly concentrations of 40–50 m^−3^), whilst rural outdoor concentrations were very low (typically 1–2 m^−3^ h^−1^). Whilst the approach shows great potential for high resolution data generation, further development is required for spectroscopic analysis and hence chemical confirmation of visual microplastic identification.

**Supplementary Information:**

The online version contains supplementary material available at 10.1007/s11356-022-24935-0.

## Introduction

Microplastics have been identified in many environments, from sediments in the deep ocean (van Cauwenberghe et al. 2013) to surface snows on Tibetan Plateau glaciers (Zhang et al. 2021), whilst their presence has also been reported in organisms from zooplankton (Cole et al. 2013) and earthworms (Huerta Lwanga et al. 2016) to cetacaeans (Zhu et al. 2019). They have been recorded in the organs of 198 fish species (Sequeira et al. 2020), in 90% of seabirds (Wilcox et al. 2015) and in human blood, lung and placental tissue (Ragusa et al. 2021; Amato-Lourenço et al. 2021; Leslie et al. 2022). Therefore, they are now generally considered ubiquitous. To date, much microplastic research has focussed on marine ecosystems and the scale and extent of contamination in other environmental compartments, including the atmosphere, remain poorly understood (Brahney et al. 2021). However, recently, airborne microplastics have started to receive attention for their potential role in atmospheric radiative forcing (Revell et al. 2021), but particularly with regard to human exposure and health effects (e.g. Zhang et al. 2020a; Zhang et al. 2020b).

Whilst microplastics have been identified in the atmosphere of remote locations (Allen et al. 2019; Bergmann et al. 2019), elevated concentrations have been reported in many urban areas (Dris et al. 2016; Klein and Fischer 2019; Li et al. 2020). For example, deposition of greater than 1000 microplastics m^−2^ day^−1^ was recorded both outdoors in London (Wright et al. 2020) and in indoor locations in Shanghai (Zhang et al. 2020b), whilst airborne concentrations of over 2500 m^−3^ have been reported at an urban roadside site, also in London (Levermore et al. 2020). However, direct comparisons between studies are complicated by the presentation of data as ‘total microplastics’ or ‘fibre-only’ values or in different units, with passive deposition data typically being reported in units of m^−2^ day^−1^ whilst actively captured atmospheric microplastics are recorded as numbers m^−3^ (Jenner et al. 2021).

Whilst sources, such as vehicle tyre-wear and the degradation of larger plastic debris, contribute to the outdoor atmospheric microplastic load (Brahney et al. 2021), additional sources are located indoors, including the release of fibres from clothing, carpets and other fabrics, and domestic activities such as laundry and mechanised clothes drying (Kapp and Miller 2020; Sobhani et al. 2020; Tao et al. 2022). As a result, some reported indoor concentrations of airborne microplastics are very high although spatial and temporal variability within and between sampling locations is considerable (Li et al. 2020; Zhang et al. 2020b; Jenner et al. 2021; Liao et al. 2021). As people tend to spend up to 90% of their time indoors (Jenner et al. 2021), some recent studies have focussed on airborne and depositing concentrations of microplastics in differing indoor environments (e.g. office, bedrooms) as well as comparisons of these with outdoor concentrations. In general, variability between, and within, indoor sampling locations has been found to be high, and considerably higher in homes (by up to 45 times) than in outdoor spaces (Jenner et al. 2021). A further factor affecting between-site variability relates to air movement. In outdoor locations airborne microplastic concentrations may be considerably altered as a result of dilution by wind (Jenner et al. 2021), whilst air movement indoors either by mechanical means, such as air conditioning, or by the movement of people, may re-entrain microplastics from where they have been deposited onto surfaces, back into the atmosphere (Prata et al. 2020a; Zhang et al. 2020b). Therefore, given rapidly increasing concerns regarding airborne microplastics (e.g., Gasperi et al. 2018; Prata 2018; Rist et al. 2018; Wang et al. 2021), there is a need to determine how airborne microplastic concentrations, across a range of particle sizes, vary on short timescales. These data would enable better estimates of temporal variability and the scale and duration of elevated concentration episodes.

Whilst studies have considered microplastic concentrations on a daily basis and compared weekdays and weekends (Zhang et al. 2020b), sampling durations are typically coarse (See review in Wright et al. 2021), and to our knowledge, there have been no assessments of airborne microplastics on an hourly timescale which would provide the high-resolution temporal data required to determine diurnal patterns. The aim of this study was to undertake a ‘proof-of-concept’ investigation into the potential viability of using an established atmospheric particulate monitoring technique to determine hourly concentrations of airborne microplastics within both indoor and outdoor environments. Here, we present the results of this investigation and provide suggestions for its further development and improvement.

## Materials and methods

The Burkard spore trap is an active volumetric air sampler that has been widely used for the monitoring of aerobiological particulates such as spores and pollen (Rantio-Lehtimäki et al. 1994; Sterling et al. 1999), but also for urban anthropogenic particles and associated pollutants (Battarbee et al. 1997; Hutton and Williams 2000). Air is drawn through a 14 mm × 2 mm orifice at a rate of 10 L min^−1^ (0.6 m^3^ hour^−1^) where suspended particles impact onto an adhesive-coated tape fixed to a clockwork-driven rotating drum moving at a rate of 2 mm hour^−1^. A period of a week may be sampled on a single 336 mm length of exposed tape which may be cut into daily sections (or other lengths as required) for analysis. The direction of the sampling orifice may be fixed or allowed to rotate to face into the wind by means of a vane attached to the sampling head. Internally, the trap is constructed entirely of metal and is simple to clean, reducing the risk of contamination from within the sampler itself. The air intake is powered by a 12 V car battery making the whole sampling system reasonably portable. The battery is usually sufficient to power the system for a full week whilst the powered intake is also quiet making it appropriate for residential sampling. Sampling efficiencies for airborne particulates greater than 5 µm are reported as over 90% (Razmovski et al. 1998; Levetin et al. 2000), although efficiencies decline for particle sizes below this and Long (1998) reported that collection efficiencies for the Burkard Trap fall below 50% for particles smaller than 2.8 µm.

A Burkard Trap was placed outside the North West Wing of University College London (UCL) (51° 31′ 27.95″ N; 0° 08′ 04.21″ W) facing the adjacent Gower Street (kerbside 3.5 m away) between 22^nd^ and 29^th^ March 2021 during the UK’s first national lockdown for the coronavirus pandemic. This sampling was repeated in-between lockdowns (7^th^–14^th^ June 2021) at which time Burkard Traps were also simultaneously located indoors within the North West Wing (foyer of the Department of Geography, UCL), as well as indoors and outdoors at a private residence in a rural location on the south-eastern edge of the village of Wadhurst, East Sussex (51° 03′ 34.97″ N; 0° 20′ 45.02″ E). Neither of the indoor locations possessed air conditioning, but both were sited in areas (e.g. foyer and hallway) where people were most likely to pass by. For these preliminary experiments, the Burkard Traps were positioned on the ground giving a height for the intake orifice of 0.5 m. Central London in the March sampling week had a mean temperature 8.9 °C and a mean wind speed of 4 m s^−1^. In the June sampling week, the mean temperature for central London was 19.7 °C, with a mean wind speed of 3.5 m s^−1^, whilst in East Sussex, these values were 17.6 °C and 2.7 m s^−1^ respectively. Hence, meteorological conditions were similar for both periods. No rain was recorded in either location during these sampling weeks (Weather Underground 2022).

Melinex® tape, cleaned to remove any particulate matter prior to exposure, was fixed to ethanol-cleaned drums for each Burkard trap. Melinex is a polyester film, but no suitable non-plastic alternative has yet been identified as an appropriate substitute. The tape was fixed to the drum at the start/end with double-sided tape and coated in silicone vacuum grease (Glisseal®) (Levetin 2004) to capture the airborne particles drawn through the orifice. Sampling began and ended on the Monday of each week, although at slightly different times in London and Wadhurst due to the time required to travel between the two. Following exposure, the tapes were cut with a scalpel into 48 mm (24 h) sections and the ends fixed to glass microscope slides using double-sided tape. All tapes and slides were covered prior to, and following, any handling to avoid contamination and all utensils and surfaces used in the handling and cutting of the tapes were also cleaned prior to each use. All sample handling was undertaken in a laboratory room with minimal air flow and human traffic, whilst a cotton laboratory coat was worn at all times to cover clothing. A sample blank, comprising a cleaned microscope slide coated with silicone grease in the same way as the Melinex tape, was left exposed in the laboratory for 1.5 h during analysis, representing the time that any tapes or slides were uncovered. A single fibre was observed on this blank slide although, for the reasons described below, its chemical composition was not confirmed spectroscopically. Either way, it represented a low level of potential contamination.

Suspected microplastic particles on the 48 mm lengths of tape representing each day were counted under a dissecting microscope at 40 × magnification. The tapes were counted chronologically using a 2 mm grid to allocate particulates to hour-long periods across the whole width of the exposed tape (14 mm). Visual microplastic identification followed the criteria used in microplastics studies from across a broad range of environments (Hidalgo-Ruz et al 2012; Turner et al. 2019; Prata et al. 2020b). Briefly, fibres are defined as equally thick throughout their length, and without tapered ends, fragments are flattened, irregular shaped and/or shard like, foams have a sponge-like texture and granules are spherical or rounded. None of these morphologies should have any visible organic or cellular structure. Size, colour and shape of each suspected microplastic particle were recorded. Size was measured using an eye-piece graticule although the microscope magnification precluded the analysis of particles below 25 µm. Fibres that were observed to overlap hours were allocated to the hour they were most contained within. Spectroscopic methods (e.g. micro-Fourier Transform Infra-Red microscopy (µFTIR); Raman) were not used in this preliminary study to chemically confirm the presence of microplastics as the Melinex tape and /or silicone adhesive coating would not be the right substrate for spectroscopic analysis of plastic particles and could be problematic for accurate comparison with plastic polymer spectra libraries. This is an area identified as a priority for future development, but as a result, microplastic numbers may be over-estimates due to visual false positives.

## Results and discussion

**Fig. 1:**
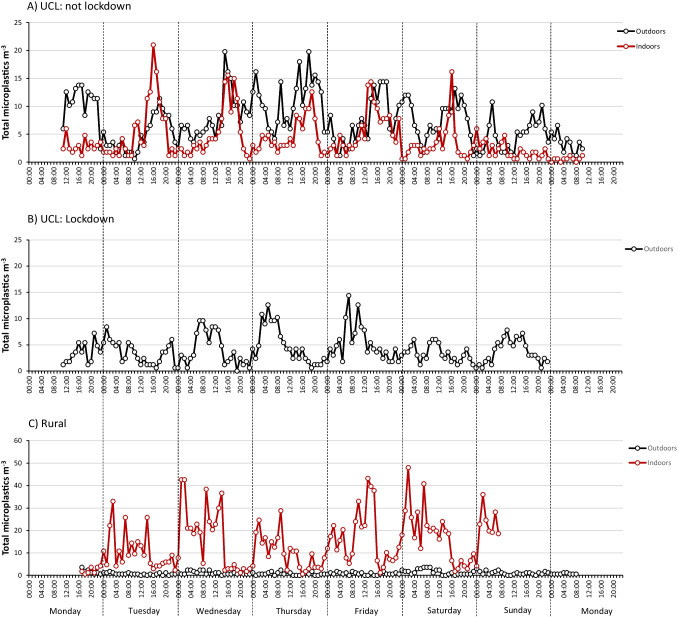
Hourly resolution of total airborne microplastics at **A** indoors and outdoors at University College London (UCL) in central London in-between coronavirus lockdowns, **B** outdoors at UCL during coronavirus lockdown and **C** indoors and outdoors at a private residence in Wadhurst, East Sussex

The hourly microplastic concentrations for each of the five sampling periods are presented in Fig. 1. Microplastics were recorded in almost every hour in each location. The records at the urban outdoor site in March (lockdown) (Fig. 1B) and the indoor rural site (Fig. 1C) were slightly curtailed due to battery failure.

### Diurnal and weekly patterns

There are clear diurnal patterns at each sampling location. At UCL in central London in June (between lockdowns) (Fig. 1A), the general weekday pattern for both indoor and outdoor locations followed similar trends with the lowest concentrations in the early hours of each day, increasing from around 0900 to peak concentrations of 15–20 m^−3^ in the early evening (ca. 1800). The outdoor sampler was located just 3.5 m from Gower Street (A400) which is a busy thoroughfare for vehicles. Approximately 160 m to the north, Gower Street joins Euston Road (A501) which is part of central London’s inner ring road and forming part of the city’s vehicle congestion charge boundary. The UK Department for Transport road traffic statistics for Gower Street for selected days between 2001 and 2017 indicate a peak in the numbers of ‘all motor vehicles’ (i.e. the total of all counted vehicles excepting bicycles) at 0800–0900 in the morning and then again at 1700–1800 in the evening with lower vehicle numbers in-between (Department for Transport 2021). The entrance to Euston Square underground station is also 130 m to the north. In years prior to lockdown, Euston Square recorded around 50,000 pedestrian combined entries and exits each weekday with an annual total exceeding 14 million in 2019 (Transport for London 2020). Transport for London ‘crowding’ statistics for the London Underground network reported at 15-min intervals for selected days show that for Euston Square, peak passenger entrances/exits occur between 0830–0900 and 1730–1800 (Transport for London 2017).

The morning increase in microplastic concentration may therefore reflect increased vehicle and pedestrian movement outdoors either entraining microplastics into the air and/or shedding from outdoor pedestrian clothing (Liu et al. 2019), whilst the evening peak may result from a similar movement of people and vehicles returning home. Corrosion and mechanical abrasion of vehicle tyres (Evangeliou et al. 2020), the use of polymer modified bitumen (Vogelsang et al. 2018) and markings in road paint (Burghardt et al. 2022) have all been considered potential microplastic sources from road use. At weekends in the outdoor location, concentrations were generally lower throughout, whilst peaks of approximately 10 m^−3^ occurred both in the early morning and late evening, probably reflecting lower vehicle and pedestrian numbers. Indoors, much lower concentrations were recorded throughout Saturday and Sunday. This is to be expected as the UCL Department of Geography was closed to most people on these days. Concentrations at both locations were broadly similar through the week except for a few notable occasions. Outdoor concentrations were considerably higher at the start of the sampling period on Monday afternoon and again between 2300 and 0300 on Wednesday-Thursday and Friday-Saturday nights whilst indoor concentrations were briefly very high at around 1600–1700 on Tuesday which may relate to the increased movement of people leaving the building and passing close to the sampler at the end of the day. During the coronavirus lockdown in March 2021 (Fig. 1B), the outdoor daily patterns for central London appeared to be different. Microplastic concentrations were generally lower throughout (more than 60% lower for greater than 40% of the time) and were again the lowest overnight. Daily peak concentrations were lower (10–15 m^−3^) and also earlier in the day on Tuesday to Thursday (0600–1200), whilst weekend concentrations (Saturday only) appeared to peak over the middle of the day (1000–1600). We speculate that these results may reflect reduced vehicle and pedestrian numbers during the pandemic lockdown.

In the rural location (Fig. 1C), outdoor microplastics concentrations were very low, less than 4 m^−3^ in any hour and typically 1–2 m^−3^. There was no discernible diurnal or weekly pattern. The nearest main road, the B2099, is 160 m from the sampling location and links local residential areas to larger arterial roads. Hence, the collection of re-entrained particles from this source is unlikely. Indoor microplastics at this location showed a curious diurnal pattern with concentrations increasing rapidly in the early hours on most days and remaining elevated until the mid to late afternoon. The lowest concentrations occurred daily from this point through to midnight. Weekend (Saturday only) and weekday diurnal patterns were similar. Indoor patterns likely reflected the movement of people within the residence. At this time during the pandemic, many people remained working from home even though the full lockdown was not in place during the sampling period. Prolonged periods of movement within the home may therefore be expected although we cannot explain why high concentrations occurred between 0200 and 0600. No windows were open near the sampler over these time periods; there were no pets and no artificial air conditioning in operation. Further work would be required to confirm these temporal patterns. The indoor peak concentrations of 40–50 m^−3^ were the highest of any location and occurred at various times of day.

### Indoor/outdoor comparisons

**Fig. 2:**
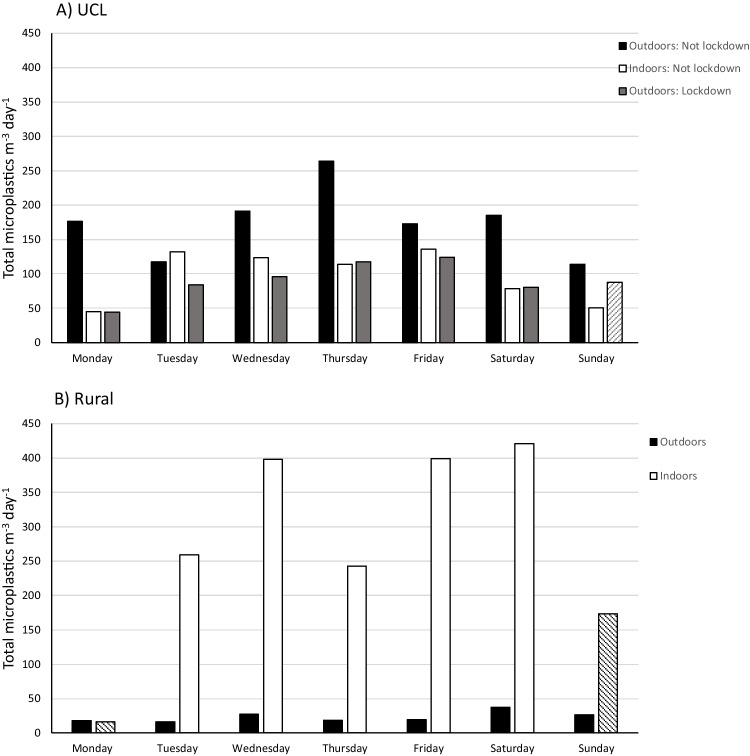
Indoor and outdoor daily total airborne microplastic concentrations recorded at **A** UCL in central London and **B** a private residence in Wadhurst, East Sussex

Whilst indoor and outdoor microplastic concentrations at UCL in central London followed similar diurnal patterns and were of similar magnitude, those at the rural location differed greatly. When indoor concentrations were at their lowest, they were comparable with those outdoors. By contrast, at their peak, indoor microplastic concentrations exceeded those recorded at the same time outdoors by over a factor of 70 (Fig. 1C). Daily values (Fig. 2) showed indoor concentrations reaching 400 m^−3^ day^−1^ on three occasions (Fig. 2B) with a mean of 344 m^−3^ day^−1^ (includes only days with complete data—Tuesday to Saturday). This compares with 23 m^−3^ day^−1^ for the rural outdoor site and 106 m^−3^ day^−1^ for the urban indoor location, highlighting the elevated airborne concentrations in residential indoor environments.

**Fig. 3:**
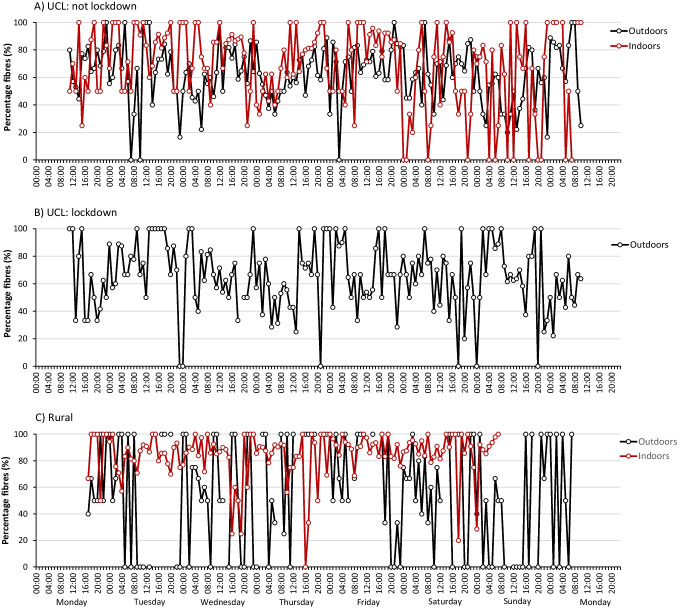
Hourly data of fibres as a percentage of total microplastics at **A** indoors and outdoors at University College London (UCL) in central London in-between coronavirus lockdowns, **B** outdoors at UCL during coronavirus lockdown and **C** indoors and outdoors at a private residence in Wadhurst, East Sussex

Fibres dominated particle morphologies in all locations representing 77% of all microplastics in the central London indoor location and 64% and 65% outdoors in the lockdown and non-lockdown periods respectively (Supplementary Information Figure S1; Fig. S2). Fragments comprised 22% of the urban indoor microplastics and 35% and 34% outdoors, in lockdown and non-lockdown weeks respectively. Granules formed the remainder (0.7–1.2%). In the rural location, fibres were again dominant, comprising 88% indoors and 58% outdoors. Fragments represented 12% and 41% of rural indoor and outdoor microplastics respectively with granules less than 1% in both. These data are consistent with many other studies which also report fibres and fragments as the most common morphologies in airborne samples. Indeed, some studies on airborne microplastics only report data on fibres (e.g. Dris et al. 2016; Tao et al. 2022). In Paris, France, and in the Humber region of the UK, fibres represented more than 90% of airborne microplastics (Dris et al. 2015; Jenner et al. 2021), around 80% in Dongguan, southern China (Cai et al. 2017) and 67% in Shanghai (Liu et al. 2019). By contrast, fragments comprised 95% of airborne microplastics in Hamburg, Germany (Klein and Fischer 2019), 83–94% in Wenzhou (Liao et al. 2021), and 87% in Aarhus (Vianello et al. 2019). Foam and film microplastics were not observed on the Burkard Trap tapes in any of the sampling locations. This is, again, similar to the results of other studies (Dris et al. 2015; Cai et al. 2017; Klein and Fischer 2019), although these particle types were identified in airborne samples from London (Wright et al. 2020). Further work is required to determine whether these morphologies are simply much less abundant in the atmosphere or whether their collection efficiencies are lower for the Burkard Trap. There was no diurnal pattern to microplastic morphologies from the hourly data (Fig. 3) although the high numbers of hours where fibres represented 100% of the microplastics highlights their morphological dominance. At the indoor rural location in particular, fibres represented over 80% of the identified microplastics for over 76% of exposure hours.

**Fig. 4:**
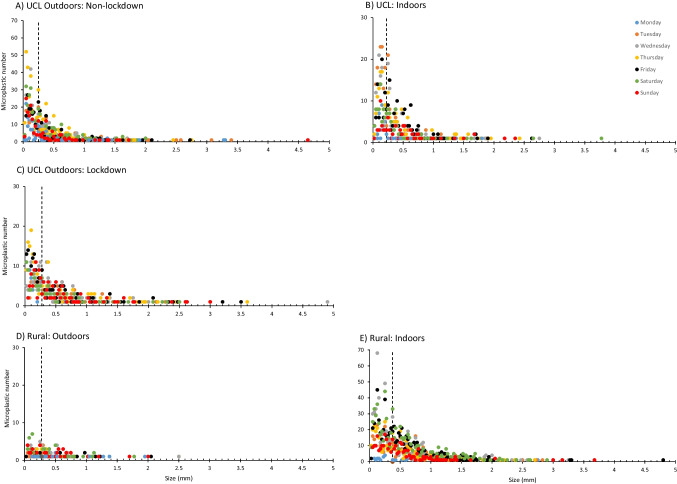
Measured sizes of airborne microplastics at **A** UCL outdoors and **B** UCL indoors in-between lockdowns, **C** UCL outdoors during lockdown, **D** outdoors and outdoors at a private residence in Wadhurst, East Sussex. Only those measured as < 5 mm are shown. Days of the week are presented as different colours and the vertical dotted line represents the 50% size-class boundary in each location

**Fig. 5:**
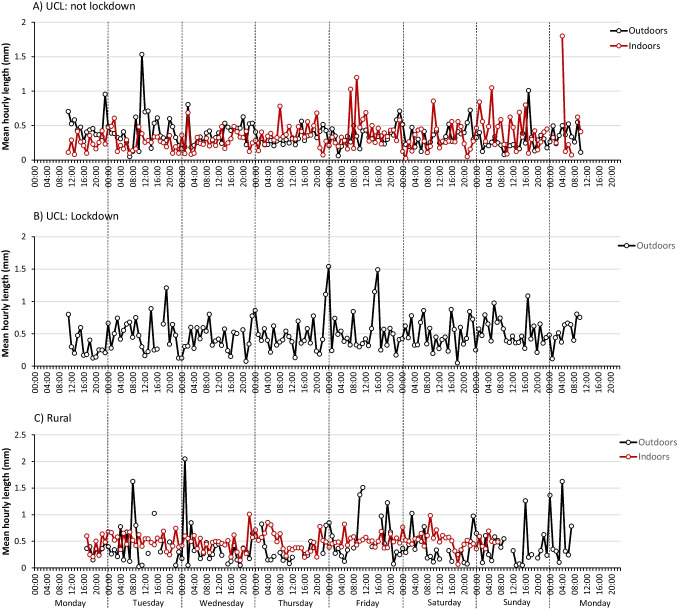
Mean hourly length data for microplastics at **A** indoors and outdoors at University College London (UCL) in central London in-between coronavirus lockdowns, **B** outdoors at UCL during coronavirus lockdown and **C** indoors and outdoors at a private residence in Wadhurst, East Sussex

In addition to morphological class, the colour and maximum dimension were also recorded for each of the 7714 microplastic particles identified under the light microscope although maximum length of fibres were sometimes difficult to assess due to their twisted nature in what have been referred to elsewhere as ‘fibre bundles’ (Rochman et al. 2019) (see Supplementary Information Figure S2). Size distributions for each of the five sampling weeks are shown in Fig. 4 as the frequency of microplastic particles in contiguous 25 µm size classes, coloured according to their day of capture. Size distributions at all locations were similar with the greatest abundance in the smaller fractions as observed in many other studies (Wright et al. 2020; Beaurepaire et al. 2021; Jenner et al. 2021; Liao et al. 2021). As expected, there was no distinction between sampling days or between weekdays and weekends (Fig. 4; Supplementary Information Figure S3). In all urban sampling weeks (indoor and outdoor) and at the rural outdoor location, 50% of the recorded microplastics were less than 225–275 µm (Fig. 4). At the rural indoor (residential) site, 50% of recorded microplastics were less than 375 µm. As with the morphological characteristics, there was no diurnal or weekly pattern in particle size at any of the sampling locations (Fig. 5). At the rural site, the largest microplastics tended to be found outdoors (Fig. 5C), but they were more evenly distributed in the urban location (Fig. 5A). Measuring the largest dimensions of microplastics clearly has a bias towards fibres which represented the largest particle class in each location. Although there is considerable scatter within the full dataset (769 paired hourly mean observations), this resulted in a significant relationship between the hourly percentage of microplastics that were fibres and the mean length of microplastics within that hour (Supplementary Information Figure S4) $\left(r=0.456;\;N=769;\;p<0.001\right)$.

One limitation of our current methodology is the lower cut-off size boundary of 25 µm which precludes accurate recording and enumeration of the smallest airborne microplastics which relate to the inhalable (< 10 µm; PM10) and respirable (< 2.5 µm; PM2.5) fractions (Li 1994; Liao et al. 2021) and which we assume will be even more abundant. However, recent reviews of atmospheric microplastic analysis and occurrence (Beaurepaire et al. 2021; Wright et al. 2021) indicate that other methodologies have similar lower boundary limits with most having a lower cut-off size limit of 10 – 25 µm (i.e. larger than the inhalable size range). Whilst our lower cut-off is defined by the magnification of the microscope used for identification, µFTIR analysis is also limited to particles larger than 10 µm and so the size limits in these other studies may, at least in part, be driven by the spectroscopic methods employed for chemical identification. As regards maximum dimensions, we noted no marked cut-off or hiatus in any of our size distributions at 2 mm, which is the vertical dimension of the Burkard trap intake orifice. Whilst we acknowledge the limitation of our datasets to date, we conclude from this preliminary investigation that the orifice dimension would not seem to be a significant barrier to sampling within the microplastic size range. It is also interesting to note that whilst most of our sampling locations recorded airborne microplastics across the size spectrum available by our methodology (25 µm–5 mm), at the rural outdoor site the largest particle recorded was a 2.5 mm fibre (Fig. 4D). This smaller outdoor size range may be due to a greater distance from potential sources such as roads or transfer from indoor locations.

Dominant colours of microplastics on the Burkard tapes from all locations were transparent (46–66%) and blue (12–32%) followed by yellow (4–22%) and red (2.3–11%) with many other colours in low numbers (Supplementary Information Figure S5). Although care is required in considering these data, as brighter colours may be more easily observed under the microscope leading to a bias in their favour when compared to white and transparent microplastics (Hartmann et al. 2019), the overwhelming dominance of transparent and blue colours in our samples would suggest any bias is likely to be minor. The dominance of blue fibres at the UCL indoor location may be a result of the preference for blue furniture fabrics throughout the Department. There were no significant differences between days of the week or locations, although blue/transparent totals were higher in the indoor locations (84–90%) compared with outdoors (61–71%). Some studies have indicated that microplastic colour may be related to polymer-type or sources. For example, Rocha-Santos and Duarte (2015) suggest transparent microplastics may be linked to polypropylene, whilst blue colours could be linked to hygiene products. However, chemical determination using techniques such as Fourier-Transform Infra-Red microscopy (µFTIR) or Raman spectroscopy is required for more definitive source apportionment. Further development is required to include chemical identification in our method for hourly microplastic sampling as the absence of such analysis currently increases the likelihood of false-positive identifications.

## Conclusions and suggestions for further study

To our knowledge, these are the first reported data for airborne microplastics at an hourly resolution. However, whilst the use of Burkard Traps is well established for aerobiological particles, this approach for microplastics is still experimental. Despite current limitations (lack of chemical analysis; limited data sets and locations), the data produced appear to show explainable and repeatable diurnal patterns and we believe the approach is therefore worthy of further research and development.

In both indoor and outdoor urban sampling locations in central London, weekday patterns appear to reflect vehicle and pedestrian movement, increasing from the morning through to a peak in the late afternoon. The close relationship here, between indoor and outdoor patterns, was likely linked to exchange between the two locations as the indoor sampling site was close to an entrance thoroughfare whilst the lack of textiles (e.g. carpets and furnishings) in this location may reduce contributions from additional indoor sources. The residential indoor sampling site showed a different daily pattern, but concentrations were also much higher and the microplastics generally larger. This was likely due to the greater presence of, and closer proximity to, indoor sources such as carpets and other fabrics. The residential diurnal pattern and elevated concentrations may therefore be due to air movement caused by people passing near the Burkard Trap. This ‘people traffic’ may either generate new microplastics from the floor covering and/or re-entrain previously deposited microplastics derived from this and other sources. Outdoors in the rural location, air movement is required to transfer microplastics from sources to the Trap but will also dilute and disperse microplastics resulting in the much lower concentrations observed.

The effects of meteorology on microplastic sampling in outdoor locations is one aspect that requires considerable further study. As with other airborne particulates, the role, especially of wind speed and direction (transfer from sources, dilution, dispersal, re-entrainment) and wet deposition (scavenging, wash-out, removal) will be major factors in the temporal patterns of microplastic concentrations. However, the Burkard Trap approach also needs development in other aspects. A greater number of sites and locations are required to replicate diurnal patterns and to gain a greater understanding of sources. Furthermore, there is a need to investigate alternative sampling tapes and adhesives that would allow direct chemical analysis, for example by Raman or µFTIR spectroscopy. Such chemical confirmation of microscopic identification is an essential development to avoid the inclusion of false positives in assessments of airborne concentrations. The approach as presented here uses low-powered microscopy for the initial identification of microplastics and is thereby limited to particles greater than 25 µm. Further development might allow the study of microplastics down to 5 µm where the Burkard Trap retains a high sampling efficiency (Razmovski et al 1998; Levetin et al 2000) which would then allow assessment of airborne microplastics within the human inhalable size range. Whilst µFTIR spectroscopy also has size limitations, Raman spectroscopy is effective for these smaller particles and the combined approach of Burkard high-resolution sampling with higher-powered microscopy and Raman spectroscopy might therefore provide useful hourly data for human exposure studies.

In the meantime, this proof-of-concept study has shown that sampling and analysis of airborne microplastics at hourly resolution are feasible. Recent studies on both particulate and gaseous urban airborne contaminants have identified variations at very small temporal and spatial scales (e.g. Vardoulakis et al 2005; Fan et al 2021) and microplastics likely vary in a similar way. A relatively small and mobile sampling technique such as the Burkard Trap, which permits hourly resolved measurements may, therefore, make a valuable contribution to the airborne microplastic sampling ‘tool-box’ for future assessments across a range of applications. For example, apart from assessing short-term outdoor variations linked to changing wind speeds and directions, it could be used indoors for laboratory assessments of airborne contamination during analysis or to determine the scale and extent of re-entrainment by people movement within residential or commercial spaces.

## Supplementary Information

Below is the link to the electronic supplementary material.

Supplementary file1 (DOCX 3890 KB)

## Data Availability

The datasets generated during the current study are available from the corresponding author on reasonable request.

## References

[CR1] Allen S, Allen D, Phoenix VR, Le Roux G, Durántez Jiménez P, Simonneau A, Binet S, Galop D (2019). Atmospheric transport and deposition of microplastics in a remote mountain catchment. Nat Geosci.

[CR2] Amato-Lourenço LF, Carvalho-Oliveira R, Júnior GR, dos Santos GL, Ando RA, Mauad T (2021). Presence of airborne microplastics in human lung tissue. J Hazard Mater.

[CR3] Battarbee JL, Rose NL, Long X (1997). A continuous, high-resolution record of urban airborne particulates suitable for retrospective microscopical analysis. Atmos Environ.

[CR4] Beaurepaire M, Dris R, Gasperi J, Tassin B (2021). Microplastics in the atmospheric compartment: a comprehensive review on methods, results on their occurrence and determining factors. Curr Opinion Food Sci.

[CR5] Bergmann M, Mützel S, Primpke S, Tekman MB, Trachsel J, Gerdts G (2019). White and wonderful? Microplastics prevail in snow from the Alps to the Arctic. Sci Adv.

[CR6] Brahney J, Mahowald N, Prank M, Cornwell G, Klimont Z, Matsui H, Prather KA (2021). Constraining the atmospheric limb of the plastic cycle. Proc Nat Acad Sci.

[CR7] Burghardt TE, Pashkevich A, Babić D, Mosböck H, Babić D, Żakowska L (2022). Microplastics and road markings: the role of glass beads and loss estimation. Transport Res d: Transp Environ.

[CR8] Cai L, Wang J, Peng J, Tan Z, Zhan Z, Tan X, Chen Q (2017). Characteristic of microplastics in the atmospheric fallout from Dongguan city, China: preliminary research and first evidence. Environ Sci Pollut Res.

[CR9] Cole M, Lindeque P, Fileman E, Halsband C, Goodhead R, Moger J, Galloway TS (2013). Microplastic ingestion by zooplankton. Environ Sci Technol.

[CR10] Department for Transport (2021) Road traffic statistics for Camden. https://roadtraffic.dft.gov.uk/local-authorities/145. Accessed 22nd November 2022

[CR11] Dris R, Gasperi J, Rocher V, Saad M, Renault N, Tassin B (2015). Microplastic contamination in an urban area: a case study in Greater Paris. Environ Chem.

[CR12] Dris R, Gasperi J, Saad M, Mirande C, Tassin B (2016). Synthetic fibers in atmospheric fallout: a source of microplastics in the environment?. Mar Pollut Bull.

[CR13] Evangeliou N, Grythe H, Klimont Z, Heyes C, Eckhardt S, Lopez-Aparicio S, Stohl A (2020). Atmospheric transport is a major pathway of microplastics to remote regions. Nat Comm.

[CR14] Fan Y, Zhan Q, Tang L, Liu H, Gao S (2021). Temporal characterization of minute-level PM2. 5 variation within a local monitoring network using DWT-DTW. Build Environ.

[CR15] Gasperi J, Wright SL, Dris R, Collard F, Mandin C, Guerrouache M, Langlois V, Kelly FJ, Tassin B (2018). Microplastics in air: are we breathing it in?. Curr Op Environ Sci Health.

[CR16] Hartmann NB, Huffer T, Thompson RC, Hassellöv M, Verschoor A, Daugaard AE, Rist S, Karlsson T, Brennholt N, Cole M, Herrling MP (2019). Are we speaking the same language? Recommendations for a definition and categorization framework for plastic debris. Environ Sci Technol.

[CR17] Hidalgo-Ruz V, Gutow L, Thompson RC, Thiel M (2012). Microplastics in the marine environment: a review of the methods used for identification and quantification. Environ Sci Technol.

[CR18] Huerta Lwanga E, Gertsen H, Gooren H, Peters P, Salánki T, Van Der Ploeg M, Besseling E, Koelmans AA, Geissen V (2016). Microplastics in the terrestrial ecosystem: implications for *Lumbricus*
*terrestris* (Oligochaeta, Lumbricidae). Environ Sci Technol.

[CR19] Hutton BM, Williams DE (2000). Assessment of X-ray photoelectron spectroscopy for analysis of particulate pollutants in urban air. Analyst.

[CR20] Jenner LC, Sadofsky LR, Danopoulos E, Rotchell JM (2021). Household indoor microplastics within the Humber region (United Kingdom): quantification and chemical characterisation of particles present. Atmos Environ.

[CR21] Kapp KJ, Miller RZ (2020). Electric clothes dryers: an underestimated source of microfiber pollution. PLoS ONE.

[CR22] Klein M, Fischer EK (2019). Microplastic abundance in atmospheric deposition within the Metropolitan area of Hamburg, Germany. Sci Tot Environ.

[CR23] Leslie HA, Van Velzen MJ, Brandsma SH, Vethaak AD, Garcia-Vallejo JJ, Lamoree MH (2022). Discovery and quantification of plastic particle pollution in human blood. Environ Internat.

[CR24] Levermore JM, Smith TE, Kelly FJ, Wright SL (2020). Detection of microplastics in ambient particulate matter using Raman spectral imaging and chemometric analysis. Anal Chem.

[CR25] Levetin E (2004). Methods for aeroallergen sampling. Curr Allergy Asthma Rep.

[CR26] Levetin E, Rogers CA, Hall SA (2000). Comparison of pollen sampling with a Burkard Spore Trap and a Tauber Trap in a warm temperate climate. Grana.

[CR27] Li CS (1994). Relationships of indoor/outdoor inhalable and respirable particles in domestic environments. Sci Tot Environ.

[CR28] Li Y, Shao L, Wang W, Zhang M, Feng X, Li W, Zhang D (2020). Airborne fiber particles: types, size and concentration observed in Beijing. Sci Tot Environ.

[CR29] Liao Z, Ji X, Ma Y, Lv B, Huang W, Zhu X, Fang M, Wang Q, Wang X, Dahlgren R, Shang X (2021). Airborne microplastics in indoor and outdoor environments of a coastal city in Eastern China. J Haz Mater.

[CR30] Liu K, Wang X, Fang T, Xu P, Zhu L, Li D (2019). Source and potential risk assessment of suspended atmospheric microplastics in Shanghai. Sci Tot Environ.

[CR31] Long X (1998) Particulate air pollution in Central London: characterisation, temporal patterns and source apportionment. Unpublished PhD thesis. University College London. 277. https://discovery.ucl.ac.uk/id/eprint/10099914/

[CR32] Prata JC (2018). Airborne microplastics: consequences to human health?. Environ Pollut.

[CR33] Prata JC, Castro JL, da Costa JP, Duarte AC, Rocha-Santos T, Cerqueira M (2020). The importance of contamination control in airborne fibers and microplastic sampling: experiences from indoor and outdoor air sampling in Aveiro. Portugal Mar Pollut Bull.

[CR34] Prata JC, Castro JL, da Costa JP, Duarte AC, Cerqueira M, Rocha-Santos T (2020). An easy method for processing and identification of natural and synthetic microfibers and microplastics in indoor and outdoor air. MethodsX.

[CR35] Ragusa A, Svelato A, Santacroce C, Catalano P, Notarstefano V, Carnevali O, Papa F, Rongioletti MCA, Baiocco F, Draghi S, D'Amore E (2021). Plasticenta: First evidence of microplastics in human placenta. Environ Internat.

[CR36] Rantio-Lehtimäki A, Viander M, Koivikko A (1994). Airborne birch pollen antigens in different particle sizes. Clin Experimen Allergy.

[CR37] Razmovski V, O'Meara T, Hjelmroos M, Marks G, Tovey E (1998). Adhesive tapes as capturing surfaces in Burkard sampling. Grana.

[CR38] Revell LE, Kuma P, Le Ru EC, Somerville WR, Gaw S (2021). Direct radiative effects of airborne microplastics. Nat.

[CR39] Rist S, Almroth BC, Hartmann NB, Karlsson TM (2018). A critical perspective on early communications concerning human health aspects of microplastics. Sci Tot Environ.

[CR40] Rocha-Santos T, Duarte AC (2015). A critical overview of the analytical approaches to the occurrence, the fate and the behavior of microplastics in the environment. Trends Anal Chem.

[CR41] Rochman CM, Brookson C, Bikker J, Djuric N, Earn A, Bucci K, Athey S, Huntington A, McIlwraith H, Munno K, De Frond H (2019). Rethinking microplastics as a diverse contaminant suite. Environ Toxicol Chem.

[CR42] Sequeira IF, Prata JC, da Costa JP, Duarte AC, Rocha-Santos T (2020). Worldwide contamination of fish with microplastics: a brief global overview. Mar Pollut Bull.

[CR43] Sobhani Z, Lei Y, Tang Y, Wu L, Zhang X, Naidu R, Megharaj M, Fang C (2020). Microplastics generated when opening plastic packaging. Sci Rep.

[CR44] Sterling M, Rogers C, Levetin E (1999). An evaluation of two methods used for microscopic analysis of airborne fungal spore concentrations from the Burkard Spore Trap. Aerobiol.

[CR45] Tao D, Zhang K, Xu S, Lin H, Liu Y, Kang J, Yim T, Giesy JP, Leung KM (2022). Microfibers released into the air from a household tumble dryer. Environ Sci Technol Lett.

[CR46] Transport for London (2020) Station Usage Data. Usage Statistics for London Stations, 2019. http://crowding.data.tfl.gov.uk/Annual%20Station%20Counts/2019/AnnualisedEntryExit_2019.xlsx Accessed 1st Feb 2022

[CR47] Transport for London (2017) London Underground Crowding data. http://crowding.data.tfl.gov.uk/ Accessed 22nd November 2022

[CR48] Turner S, Horton AA, Rose NL, Hall C (2019). A temporal sediment record of microplastics in an urban lake, London, UK. J Paleolimnol.

[CR49] Van Cauwenberghe L, Vanreusel A, Mees J, Janssen CR (2013). Microplastic pollution in deep-sea sediments. Environ Pollut.

[CR50] Vardoulakis S, Gonzalez-Flesca N, Fisher BE, Pericleous K (2005). Spatial variability of air pollution in the vicinity of a permanent monitoring station in central Paris. Atmos Environ.

[CR51] Vianello A, Jensen RL, Liu L, Vollertsen J (2019). Simulating human exposure to indoor airborne microplastics using a Breathing Thermal Manikin. Sci Rep.

[CR52] Vogelsang C, Lusher A, Dadkhah ME, Sundvor I, Umar M, Ranneklev SB, Eidsvoll D, Meland S (2019) Microplastics in road dust–characteristics, pathways and measures. NIVA-rapport. https://toi.brage.unit.no/toi-xmlui/handle/11250/2670146

[CR53] Wang Y, Huang J, Zhu F, Zhou S (2021). Airborne microplastics: a review on the occurrence, migration and risks to humans. Bull Environ Contamin Toxicol.

[CR54] Weather Underground (2022) https://www.wunderground.com/ Accessed 1^st^ February 2022

[CR55] Wilcox C, Van Sebille E, Hardesty BD (2015). Threat of plastic pollution to seabirds is global, pervasive, and increasing. Proc Nat Acad Sci.

[CR56] Wright SL, Ulke J, Font A, Chan KLA, Kelly FJ (2020). Atmospheric microplastic deposition in an urban environment and an evaluation of transport. Environ Internat.

[CR57] Wright SL, Gouin T, Koelmans AA, Scheuermann L (2021). Development of screening criteria for microplastic particles in air and atmospheric deposition: critical review and applicability towards assessing human exposure. Microplast Nanoplast.

[CR58] Zhang J, Wang L, Kannan K (2020). Microplastics in house dust from 12 countries and associated human exposure. Environ Internat.

[CR59] Zhang Q, Zhao Y, Du F, Cai H, Wang G, Shi H (2020). Microplastic fallout in different indoor environments. Environ Sci Technol.

[CR60] Zhang Y, Gao T, Kang S, Allen S, Luo X, Allen D (2021). Microplastics in glaciers of the Tibetan Plateau: evidence for the long-range transport of microplastics. Sci Tot Environ.

[CR61] Zhu J, Yu X, Zhang Q, Li Y, Tan S, Li D, Yang Z, Wang J (2019). Cetaceans and microplastics: first report of microplastic ingestion by a coastal delphinid, *Sousa*
*chinensis*. Sci Tot Environ.

