# Supplementary material for: High temporal resolution records of outdoor and indoor airborne microplastics
Source: Environ Sci Pollut Res Int. 2023 Jan 4;30(13):39246–57. doi: 10.1007/s11356-022-24935-0 (PMC9812541; doi:10.1007/s11356-022-24935-0)

**High temporal resolution records of outdoor and indoor airborne microplastics**

Lucy C. Boakes, Ian R. Patmore, Chiara E.P. Bancone, Neil L. Rose*

Environmental Change Research Centre, Department of Geography, University College London, Gower Street, London WC1E 6BT, UK.

* author for correspondence

**Supplementary Information**

**Figure S1.** Airborne microplastic morphologies at (A) UCL and (B) the rural location at Wadhurst, East Sussex.


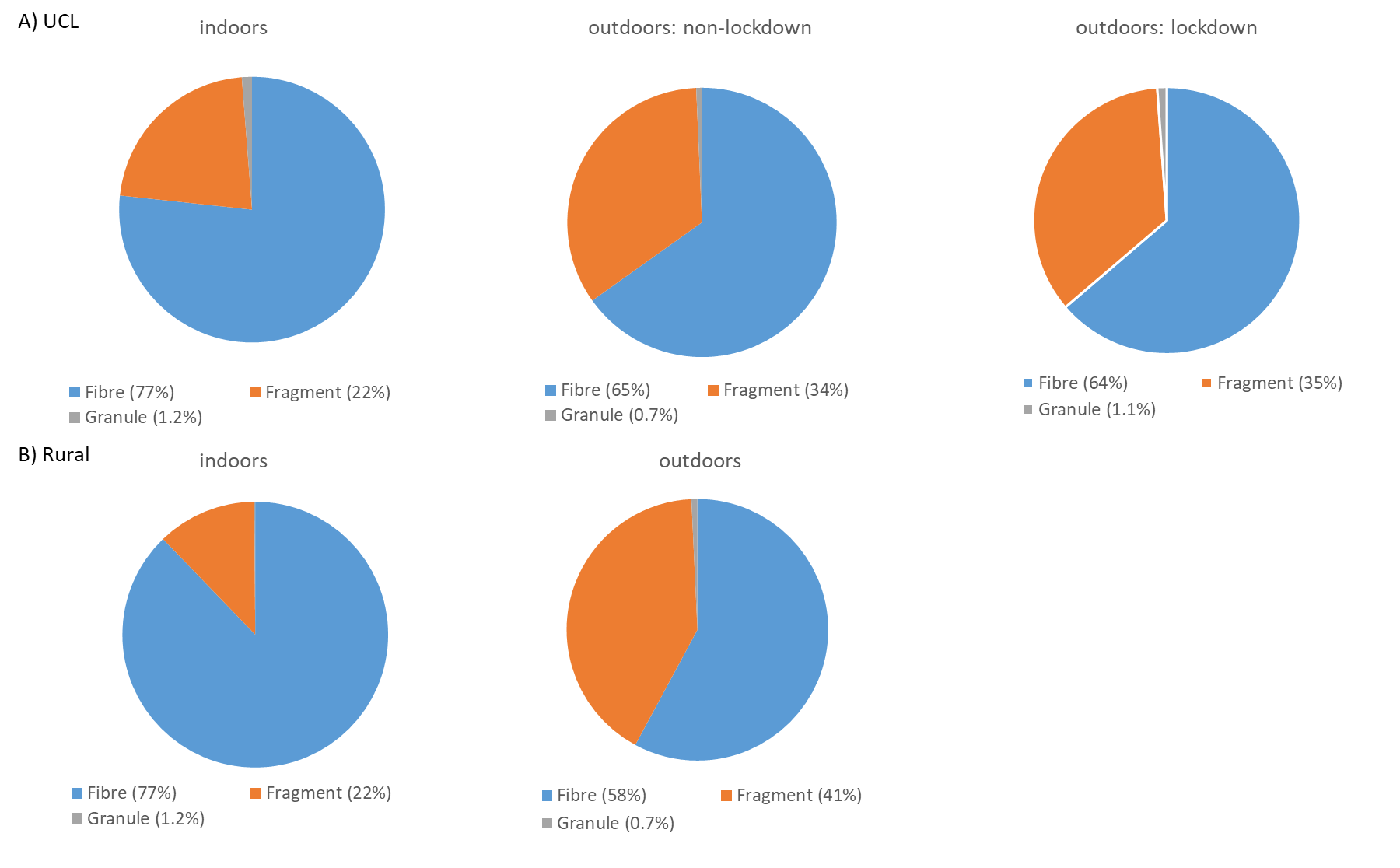


**Figure S2.** Examples of observed microplastics from the five site locations. A-E: fibres; F-H: fragments; I: a granule.

**Figure S3.** Box and whisker plot of mean hourly microplastic length for each sampling location plotted by sampling day. Boxes show the median and interquartile ranges, while the whiskers indicate 1.5x the interquartile range. Individual hours falling outside these limits are shown as dots.

**Figure S4.** Mean hourly microplastic length plotted against the percentage of microplastics within that hour that are fibres (N= 769 paired hourly mean observations). Colours represent sampling locations.

**Figure S5.** Airborne microplastic colour distributions at (A) UCL and (B) the rural location at Wadhurst, East Sussex.


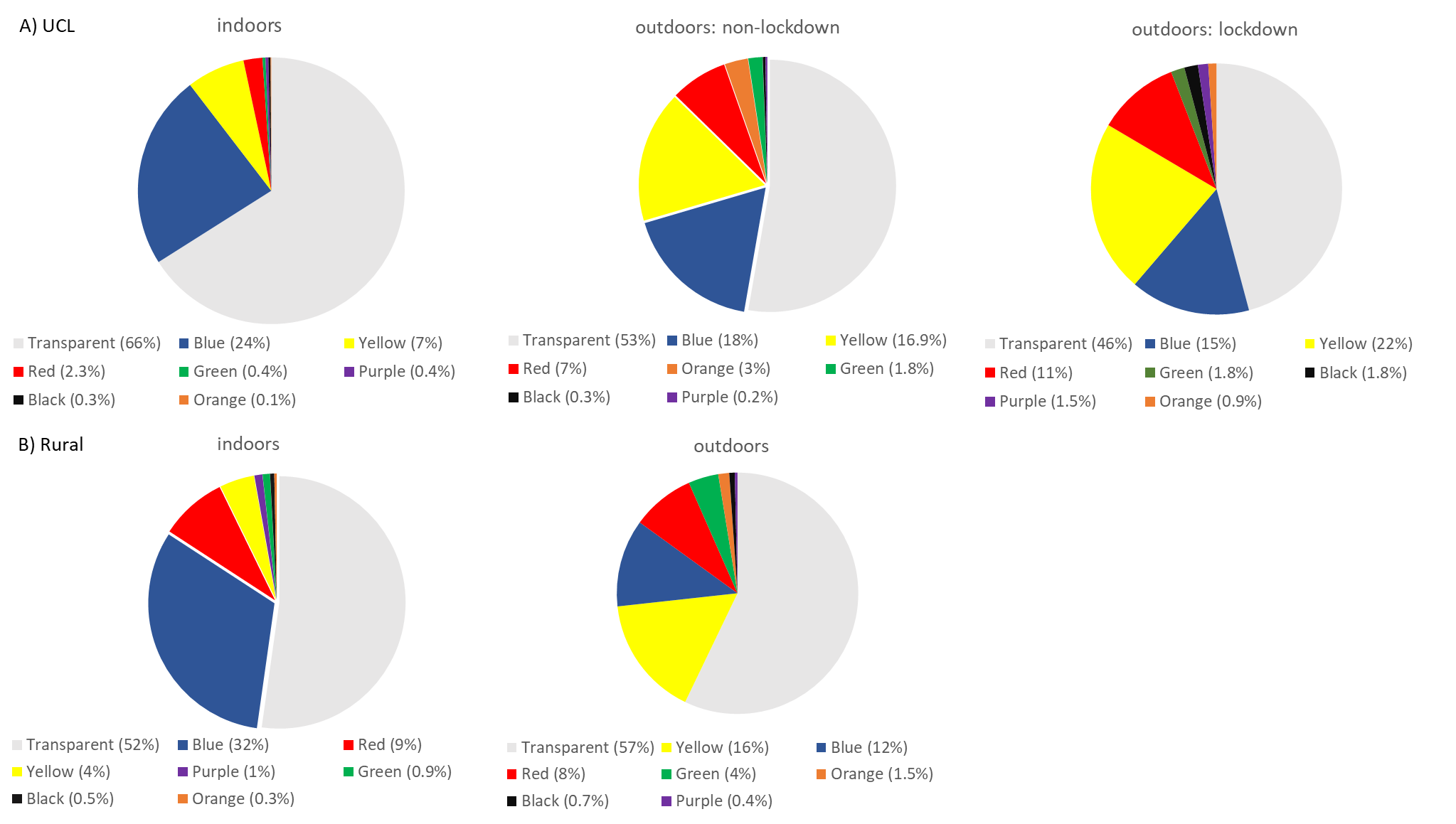

Supplement: Supplementary file 1 — Supplementary file1 (DOCX 3890 KB) [file 11356_2022_24935_MOESM1_ESM.docx]
